# Enhancing diabetes treatment by targeted nucleic acid and drug delivery using cell-penetrating peptides, peptide nucleic acids, and receptor targeting

**DOI:** 10.3389/fphar.2026.1867532

**Published:** 2026-06-18

**Authors:** Bozidarka L. Zaric, Sara Khodahemmati, Sonja S. Zafirovic, Esma R. Isenovic

**Affiliations:** Department of Radiobiology and Molecular Genetics, VINČA Institute of Nuclear Sciences – National Institute of the Republic of Serbia, University of Belgrade, Belgrade, Serbia

**Keywords:** antibody, cell-penetrating peptides, diabetes, insulin, peptide nucleic acids

## Abstract

Precise regulation of gene expression and drug delivery is required to maximize therapeutic efficacy and minimize adverse side effects. Several studies indicate a potential role for targeted gene and drug delivery in the precision therapy of type 2 diabetes and other diseases. This review summarizes recent knowledge on the cell-penetrating function and membrane-crossing roles of cell-penetrating peptides, the application of peptide nucleic acids for gene modulation, and the precise delivery of genetic material and drugs to insulin signaling and other relevant β-cell and type 2 diabetes-related genes through antibody/receptor targeting. The rationale for peptide nucleic acid-based delivery platforms is then reviewed, including their neutral charge, strong hybridization capacity, versatility, and nuclease resistance, These properties enable precise modulation of gene expression in relevant tissues for the treatment of type 2 diabetes. Finally, the review also focuses on the delivery approaches of antibody- and receptor-mediated strategies. These strategies use either whole antibodies or engineered antibody fragments and are typically used to deliver treatments to β-cells and to cells involved in metabolism.

## 1 Introduction

Hundreds of millions of people worldwide are affected by type 2 diabetes mellitus (T2D) and its related diseases and complications (Sun et al., 2022). The increased incidence of T2D is expected to raise direct care costs to over USD 845 billion by 2045. Over 90% of all diabetes diagnoses are T2D. T2D affects patients' macro- and microvasculature through hyperglycemia, obesity, and chronic inflammation (Forbes and Cooper, 2013).

Targeted pharmacotherapies can help correct the physiological changes observed in T2D while minimizing unwanted side effects. T2D may benefit from three types of directed delivery strategies: cell-penetrating peptides (CPPs), peptide nucleic acids (PNAs), and antibody-drug conjugates (ADCs). CPPs promote the intracellular transport of pharmacological cargoes across biological membranes (Guo et al., 2016; Guidotti et al., 2017), while PNAs facilitate stable, sequence-based gene silencing and knockdown (Saarbach et al., 2019; Singh and Monga, 2023). ADCs increase cell-type selectivity through receptor-mediated targeting (Ämmälä et al., 2018; Jiao et al., 2024). These platforms, used individually or in combination, could enable targeted, cell-specific modulation of T2D pathways.

The goals of directed delivery are to both increase medication efficacy and decrease unwanted side effects. CPPs enable the delivery of insulin and other drugs across oral, nasal, and pulmonary routes without invasive procedures (Khafagy et al., 2009; Patel et al., 2009; Wang et al., 2025). Trans-activator of transcription (TAT), R8-R9, penetratin, and glycosaminoglycan-binding enhanced transduction (GET) are all examples of amphipathic and cationic CPPs that enhance endosomal uptake and payload release into cells (Erazo-Oliveras et al., 2012; Guo et al., 2016). CPPs are typically used to overcome barriers to the delivery of pharmacotherapy.

In contrast, PNAs offer sequence-specific modulation of gene expression (Saarbach et al., 2019). They are synthetic analogs of DNA/RNA that can bind tightly to DNA or messenger RNA (mRNA) transcripts, thereby suppressing genetic activity (Nielsen and Haaima, 1997; Doyle et al., 2001). Moreover, compared with conventional oligonucleotides, the neutral charge of PNAs and therefore the lack of electrostatic repulsion makes them more stable in the *in-vivo* environment (Eriksson and Nielsen, 1996). Their pseudopeptide backbone also makes PNAs resistant to nuclease degradation (Shakeel et al., 2006).

Important negative regulators of insulin signaling may be targets for PNA therapies in T2D. Protein tyrosine phosphatase 1B (PTP1B) and phosphatase and tensin homolog (PTEN) are two examples. Standard antisense oligonucleotides (ASOs) targeting these genes have shown therapeutic efficacy in earlier studies (Butler et al., 2002; Zinker et al., 2002). To date, however, PNA-based therapies targeting these genes have not been experimentally tested.

Antibody- and receptor-mediated strategies facilitate drug delivery to T2D-associated cells by targeting specific cell-surface markers. In particular, therapeutics are attached to a monoclonal antibody (mAb) or a ligand that binds to a specific receptor on the target tissue (Ussar et al., 2011; Chodorge et al., 2018). Recent studies have also explored the use of ADCs and AOCs for the management of metabolic disorders (Jiao et al., 2024). For instance, Ämmälä et al. in an *in-vivo* study delivered antisense molecules directly to pancreatic β-cells, enabling suppression of gene activity through receptor-based targeting (Ämmälä et al., 2018).

While precision medicine in T2D has substantially improved with the use of novel targeted delivery systems, several barriers remain. These include adverse reactions, complex production processes, potential immune responses, and efficient intracellular trafficking of bound drugs (Jiao et al., 2024). To improve therapeutic delivery, directed delivery platforms may also be combined.

Linking CPPs to PNAs further enhances cellular uptake and therapeutic efficacy by transporting neutral PNA molecules into the cytosol and nucleus (Díaz-Mochón et al., 2005; Shiraishi and Nielsen, 2006).

**FIGURE 1:**
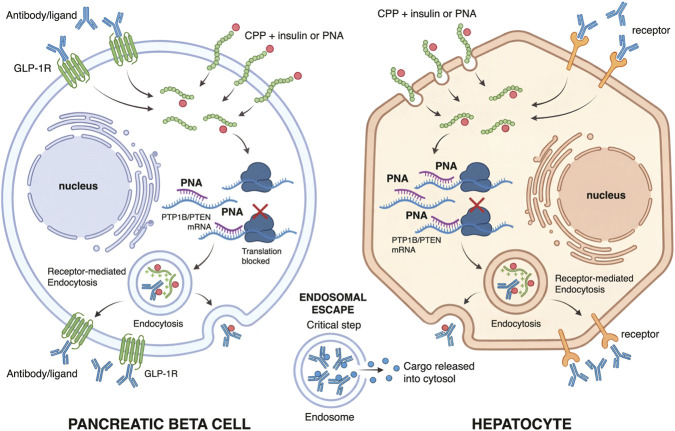
Three delivery platforms for T2D therapeutics CPPs carry cargo (insulin, PNA) across the cell membrane. PNAs hybridize with target mRNA (PTP1B, PTEN) to block translation. Antibody/ligand conjugates bind surface receptors (GLP-R on β cells) for receptor-mediated endocytosis. Inset: endosomal escape barrier.

Despite advancements in CPPs, PNAs, and antibody/receptor-based delivery systems, their application in human T2D remains at an early stage (Table 1). At present, there are no approved CPP-, PNA-, or antibody/receptor-mediated delivery systems for the treatment of T2D (Xie et al., 2020; Jiao et al., 2024). Most CPP studies have been conducted in rodents or *in vitro* epithelial models (Xie et al., 2020). Although PNAs may have potential in metabolic disease, most clinical research has focused on oncology and infectious diseases (Gupta et al., 2017). To date, no PNA trial in metabolic disease has been conducted (Figure 1).

**TABLE 1:** CPP, PNA, and antibody-based delivery platforms in early-stage development of T2D and metabolic therapies.

Platform	Clinically approved examples	Development phase/Stage	Evidence base	Administration route	Translational limitation
CPP-insulin	Rybelsus (SNAC) (Solis-Herrera et al., 2024)	Preclinical	Rodent and Caco-2 studies; 18.2% % availability in rats (Nielsen et al., 2014)	Oral/nasal/pulmonary (Khafagy et al., 2009; Kamei et al., 2013)	Low bioavailability; no human data; distinct from SNAC (Kristensen et al., 2016; Rehmani and Dixon, 2018)
CPP-GLP-1RA	Rybelsus (SNAC) (Solis-Herrera et al., 2024)	Preclinical	Penetrating increased nasal GLP-1 bioavailability to ∼15.9% in rats (Khafagy et al., 2009)	Oral/nasal (Khafagy el et al., 2009)	No clinical validation; SNAC already delivers oral semaglutides (Solis-Herrera et al., 2024)
PNA antisense (GalNAc-conjugated)	Leqvio (inclisiran) (Springer and Dowdy, 2018; Debacker et al., 2020)	Conceptual/early preclinical	GalNAc–PNA uptake and miR-122 knockdown in mice (Price et al., 2019; Kumar et al., 2023)	Parenteral	No metabolic *in vivo* efficacy model; limited development toolkit (Gupta et al., 2017; Collins and Farnsworth, 2025)
Antibody-based/receptor-mediated	None in metabolic disease; ADCs approved in oncology (Egholm et al., 1993; Metrangolo and Engelholm, 2024; Sanjanwala et al., 2025)	Preclinical	β-cell targeting studies; GLP-1R-directed ASO delivery (Ämmälä et al., 2018; Knerr et al., 2021)	Parenteral	No validated β-cell-specific target with high specificity (Collins and Farnsworth, 2025)
Integrated CPP–PNA–receptor delivery system	None	Conceptual	Individual components tested separately (Bendifallah et al., 2006; Shiraishi and Nielsen, 2011; Kumar et al., 2023)	Parenteral	No integrated *in vivo* system; combined efficacy undemonstrated

## 2 Peptide carriers for therapeutic delivery in T2D management

Cell-penetrating peptides, typically composed of 5 to 30 amino acids, can cross cell membranes and are commonly used to deliver nucleic acid or peptide therapeutics (Guo et al., 2016; Datta, 2025). They can also be combined with nanocarriers to protect cargo from enzymatic degradation. Their epithelial transfer occurs via endocytosis or, in some cases, direct translocation across the cell membrane (Guo et al., 2016).

Cell-penetrating peptides show promise for T2D therapies. In research settings, they have been used to overcome non-invasive delivery barriers for drugs such as insulin and glucagon-like peptide-1 receptor agonists (GLP-1RAs) (Khafagy et al., 2009; Korivi et al., 2021). These systems can facilitate cargo uptake into the liver, adipose tissue, and pancreas, which are key organs for metabolic homeostasis (Wancewicz et al., 2010).

By applying this design approach to PNA-based therapeutics, it may be possible to modulate molecular pathways associated with T2D, including gluconeogenesis, insulin signaling, and inflammation. However, these applications have not yet been demonstrated experimentally.

**FIGURE 2:**
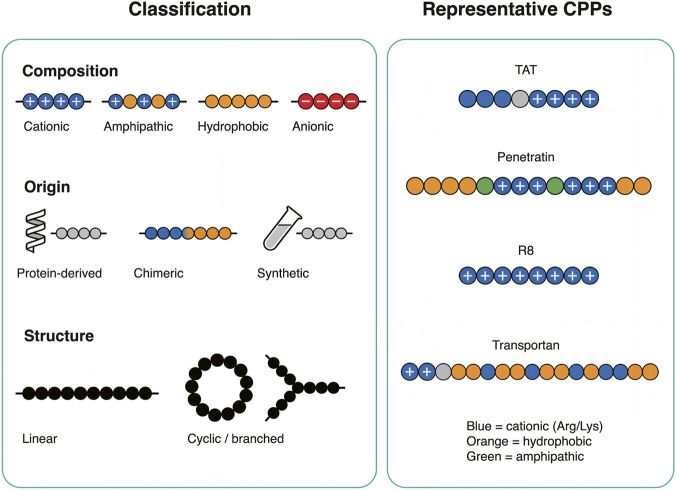
CPP classification, sequences, and structural architectures CPPs can be described by their composition (cationic, amphipathic, hydrophobic, anionic), origin (protein-derived, chimeric, synthetic), and structural features (linear, cyclic, branched). Representative sequences: TAT, penetratin, R8, Transportan.

Cell-penetrating peptides are classified according to origin, mechanisms of uptake, biological applications, and physical properties (Datta, 2025) (Figure 2). The principal compositional classes are cationic, amphipathic, and hydrophobic (Milletti, 2012). Among these, cationic CPPs are the most widely studied (Datta, 2025). Cationic CPPs include human immunodeficiency virus type 1 TAT, GET-type peptides, polyarginine Rn sequences, polylysine, and penetratin. These domains are enriched in positively charged amino acids, such as arginine, lysine, and histidine (Ruseska and Zimmer, 2020).

Cationic CPPs interact strongly, *via* electrostatic forces, with negatively charged membrane components such as glycosaminoglycans and anionic phospholipids. For the same reason, they also bind anionic therapeutic cargos, such as insulin or nucleic acids, through their positive charge (Behzadipour and Hemmati, 2024; Moreno-Vargas and Prada-Gracia, 2024). The optimal number of arginine residues for efficient translocation has been shown to be 8-10, based on studies of oligoarginines (Rn) with 4-16 residues in three cell lines (Wender et al., 2000; Futaki et al., 2001). Because arginine guanidinium groups can form bidentate hydrogen bonds together with sulfate and phosphate groups and tend to accumulate at the membrane, peptides with this number of residues can enter cells and reduce the energy barrier for translocation (Rothbard et al., 2004).

Amphipathic CPPs comprise segments that frequently assume α-helical or β-sheet conformations. These structures present hydrophilic and hydrophobic surfaces toward the membrane (Guo et al., 2016; Kalafatovic and Giralt, 2017). MAP peptides, transportan, and nuclear localization signal-containing peptides, among others, feature these structures (Guo et al., 2016). They are suitable for transporting oligonucleotides, proteins, and nanoparticles because they can associate with both lipid bilayers and aqueous environments through their hydrophobic and hydrophilic properties (Guidotti et al., 2017; Ruseska and Zimmer, 2020; Silva et al., 2022).

Hydrophobic CPPs composed mainly of nonpolar components, can diffuse across cellular membranes due to their moderate net positive charge (Milletti, 2012; Guo et al., 2016) and are commonly used in oncology and immunotherapy applications (Derakhshankhah and Jafari, 2018). Their low demonstrated toxicity and limited aggregation with nucleic acids may also make these CPPs suitable for gene delivery in metabolic disease and other applications.

A minor subclass is anionic CPPs (aCPPs)**.** At physiological pH, these CPPs are distinguished by a net-negative charge resulting from aspartic acid or glutamic acid residues (Martín et al., 2011; Reissmann and Filatova, 2021; Gori et al., 2023). Unlike cationic CPPs, they are electrostatically repelled by negatively charged components of the cell membrane (Neves-Coelho et al., 2017; Gori et al., 2023). These aCPPs are taken up by cells *via* specific amphipathic sequences or through pairing with hydrophobic domains, usually through energy-dependent endocytosis (Martín et al., 2011; Neves-Coelho et al., 2017; Ruseska and Zimmer, 2020). In chronic conditions, aCPPs may be suitable for repeated administration because of their relatively low toxicity (Ruseska and Zimmer, 2020).

Most T2D research uses cationic or amphipathic CPPs, although auxiliary modifications to aCPPs could influence cellular uptake and PNA distribution to specific organs.

Based on structure, CPPs can be categorized into linear and cyclic/branched classes (Kalafatovic and Giralt, 2017). For instance, TAT is a linear CPP well-known for its ease of synthesis, high penetration capacity, and strong membrane activity. However, linear CPPs are also prone to proteolysis, which may pose a risk factor for sensitive cargos (Grunwald et al., 2009). Unlike linear CPPs, cyclic CPPs are synthesized using more complex methods, such as head-to-tail cyclization or side-chain constraints (Kalafatovic and Giralt, 2017). Nonetheless, branched CPPs are more stable, can resist protease degradation, and are less prone to endosomal entrapment (Brock et al., 2019). Similarly, branched penetratin-based CPPs, such as penetramax dimers and trimmers have demonstrated enhanced cellular uptake and greater insulin absorption than their linear counterparts (Diedrichsen et al., 2025). These findings highlight branched and cyclic CPP architectures as the most clinically promising next-generation strategies, combining improved proteolytic stability with superior transepithelial delivery (Tietz and Cortezon-Tamarit, 2022; Diedrichsen et al., 2025).

### 2.1 Binding of cargos to CPPs and implications for T2D

The binding of cargos to CPPs can involve both covalent and non-covalent attachment. For instance, thioether, amide, or disulfide bonds can be used to attach CPPs covalently to protein drugs or peptides (Gayraud et al., 2021). Some cargos, such as nucleic acids, require modification to maintain their therapeutic activity. For example, special linkers can be added at the ends of, or within, the molecule to ensure that the genetic material can still bind effectively to its target (Bendifallah et al., 2006). Disulfide linkers are useful for preserving the integrity of CPP-cargo conjugates in extracellular environments while enabling cargo release in the reducing enviroment of the cytosol (Saito et al., 2003).

Several TAT-insulin conjugates have been designed with bifunctional cross-linkers that connect TAT thiols to insulin amines, thereby retaining insulin bioactivity and improving pulmonary uptake (Patel et al., 2009).

Non-covalent conjugates rely on non-covalent bonding mechanisms to deliver payloads to cells. Cationic CPPs and anionic cargos depend on electrostatic attraction between positively charged CPPs and negatively charged therapeutics, including insulin, GLP-1 analogs, and nucleic acids (Behzadipour and Hemmati, 2024). Hydrophobic interactions with lipid domains and hydrogen bonding also facilitate the spontaneous, non-covalent association of CPPs with conjugates (Menacho-Melgar et al., 2019; Behzadipour and Hemmati, 2024).

R6EW peptides and SAR6EW, the stearyl analog of R6, assemble with insulin at a 1:1 ratio. These assemblies depend on guanidinium-mediated hydrogen bonding and hydrophobic contacts involving tryptophan or stearyl groups, which drive membrane insertion and shield insulin from proteolysis (Rothbard et al., 2004; Zhang et al., 2015). Although non-covalent CPP conjugates are simpler to manufacture, their stability may vary (Kristensen et al., 2016), with potential implications for batch-to-batch reproducibility and large-scale production.

The density of CPPs on nanoparticle surfaces affects membrane affinity and cellular uptake across various carriers, including liposomes, solid lipid nanoparticles, and polymeric nanoparticles. In one study, chitosan or lipid nanoparticles decorated with TAT or related CPPs increased insulin endocytosis in diabetic animal models (Li et al., 2025; Wang et al., 2025).

Elevated local concentrations of cationic CPPs on PNA-loaded nanoparticles, or PNAs themselves, may enhance adhesion to β cells or hepatocytes. However, higher concentrations may also increase the probability of complement activation (La-Beck et al., 2020; Moghimi et al., 2020). They can also promote hemolysis and off-target uptake (Moghimi et al., 2020). Careful tuning of hydrophobicity and charge, supported by *in silico* predictions, may help balance an acceptable safety profile with efficient delivery (Gupta et al., 2013).

### 2.2 Cellular uptake pathways of cell-penetrating peptides: direct translocation *versus* endocytosis

**FIGURE 3:**
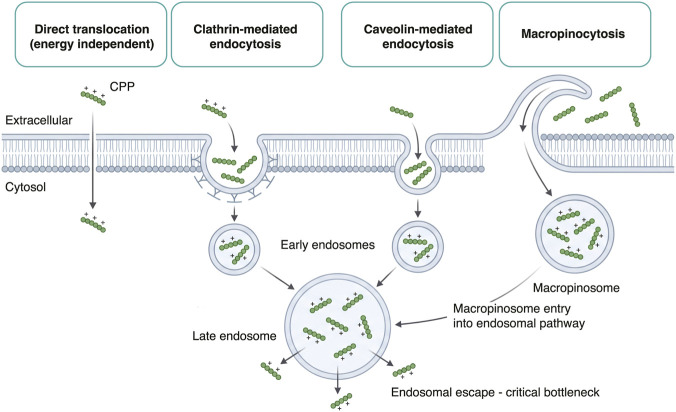
CPP cellular uptake routes and endosomal escape CPPs enter cells via four main pathways: direct translocation, clathrin-mediated endocytosis, caveolin-mediated endocytosis, and macropinocytosis. Following internalization, peptides progress through early and late endosomes. Endosomal escape presents the main challenge for CPPs to successfully enter the cytosol.

Cell-penetrating peptides cross membranes by endocytic mechanisms or direct translocation. Most systems enter cells using both routes (Figure 3) (Ruseska and Zimmer, 2020). Cationic and amphipathic CPP sequences are most frequently associated with direct translocation. Importantly, this non-endocytic process does not require the cell’s active internalization machinery (Di Pisa et al., 2015). The balance between translocation and endocytosis depends on peptide concentration and experimental conditions (Trabulo et al., 2010; Ruseska and Zimmer, 2020). Several mechanistic models have been proposed to explain direct translocation (Kauffman et al., 2015).

The inverted micelle model proposes that CPPs associate with the external surface of the plasma membrane, perturb local lipid organization, and promote inverted micelle formation (Kawamoto et al., 2011; Ruseska and Zimmer, 2020). CPPs then localize to the inner hydrophilic compartment of micelles and are released into the cytoplasm as micelle stability diminishes (Swiecicki et al., 2014). This mechanism is more likely to apply to the translocation of small peptides and less likely to apply to larger CPP-cargo conjugates (Trabulo et al., 2010; Guo et al., 2016).

Pore-formation direct-translocation models propose that CPPs integrate into the bilayer and form temporary toroidal or barrel-stave pores, especially when amphipathic helices are involved (Matsuzaki, 2019). In barrel-stave pores, peptide structures form a ring within the membrane, creating a pore that allows substances to interact with lipid tails via their hydrophobic ends and hydrophilic sides on the interior (Matsuzaki, 2019). In toroidal pores, CPPs bind to negatively charged phospholipids via electrostatic interactions, altering membrane curvature and significantly rearranging membrane components (Herce and Garcia, 2007; Herce et al., 2009). This results in bending and thinning of the cell membrane, allowing both lipid headgroups and peptides to line the pore (Alimohamadi, 2023). This can be visualized in molecular dynamics simulations (Alimohamadi, 2023). Toroidal pores act as temporary openings for small cargoes and water, and the membrane restores to its original form after the pores collapse (Herce and Garcia, 2007).

The carpet model of membrane disruption induces translocation by coating the membrane surface with high CPP concentrations, leading to detergent-like disruption (Shai, 1999; Gamage and Pan, 2025). Most therapeutic CPP systems carrying insulin, PNAs, nanoparticles, or other large cargos mainly enter cells through energy-dependent endocytosis under clinically relevant conditions (Lundin et al., 2008; Ruseska and Zimmer, 2020). For instance, endocytosis mediated by clathrin is initiated by binding of CPP-cargo complexes or cargos to extracellular receptors, after which adaptor protein-2 (AP2) is recruited (Kaksonen and Roux, 2018). AP2 recruits clathrin triskelia and assembles them into a lattice that coats the outside of membrane pits and encourages the membrane to move inward to create pits that break away from the membrane through dynamin-dependent vesicle scission (Kaksonen and Roux, 2018). The resulting ∼100–200 nm vesicles mature from early to late endosomes (Kirchhausen et al., 2014), and cargo escapes to avoid lysosomal degradation (Erazo-Oliveras et al., 2012). The cargos that are translocated through clathrin-mediated endocytosis include many CPP-insulin systems, such as TAT and R6EW derivatives, as shown in Caco-2 and nasal epithelial models (Zhang et al., 2015; Derakhshankhah and Jafari, 2018).

Caveolin-mediated endocytosis can transport cargoes to different intracellular compartments. In some cases, this pathway may even avoid rapid lysosomal degradation, which is beneficial to PNAs and other fragile cargos (Norkin and Kuksin, 2005; Kiss and Botos, 2009; Derakhshankhah and Jafari, 2018).

It is well established that transportan and other chimeric CPPs can mediate both clathrin- and caveolin-dependent endocytosis, depending on the cell type and the payload (Lundin et al., 2008; Säälik et al., 2009). Macropinocytosis is an actin-driven process that engulfs extracellular fluid (Swanson, 2008) and CPP-cargo complexes. This occurs when ruffled regions of the membrane bend back and merge to form large macropinosomes (Swanson, 2008).

Strong interactions of CPP-membrane or growth factor signaling are able to begin macropinocytosis. This mechanism is particularly important for bigger sized CPP-cargo aggregates, such as high molecular weight cargo aggregates and nanoparticles (Ruseska and Zimmer, 2020).

Charge, cargo size, hydrophobicity, cell type, formulation, and peptide concentration are all factors that influence the dominant internalization route of any CPP sequence (Kauffman et al., 2015). Depending on the uptake mechanism, cargo may remain trapped in endosomes or escape into the cytosol or the nucleus (Erazo-Oliveras et al., 2012). CPPs for antidiabetic drugs and PNAs, therefore, need to favor efficient endosomal escape to achieve high uptake (Erazo-Oliveras et al., 2012; Derakhshankhah and Jafari, 2018).

### 2.3 CPPs in antidiabetic delivery: insights from insulin, metformin, GLP-1RAs, PPAR agonists

Cell-penetrating peptides are most commonly used to formulate insulin for non-invasive, efficacious oral, nasal, or pulmonary formulations (Kamei et al., 2013). Significant differences in bioavailability have been reported when CPPs are delivered via different routes. For example, using conjugation strategies for oral delivery tends to produce only limited enhancements in effectiveness. In experiments using mice, D-penetratin–insulin showed an availability of approximately 18.2%, compared subcutaneously administered insulin (Nielsen et al., 2014). In rats, L-penetratin–insulin achieved up to 76.7% pharmacological bioavailability via the nasal route (Khafagy el et al., 2009). Pulmonary delivery is intermediate, with TAT–insulin and oligoarginine (r9)–insulin achieving 19- and 27-fold greater alveolar epithelial transport over unconjugated insulin, respectively (Patel et al., 2009). No CPP-based product delivered by any route has yet reached human trials. A further difficulty comes from anatomical variations in mucosal tissue across species, which hinder successful clinical translation. ASOs and siRNAs are the dominant approved oligonucleotide platforms. As shown by Springer and Dowdy (2018), GalNAc–siRNA conjugates effectively target and reduce gene expression in the human liver at 1–3 mg/kg (Springer and Dowdy, 2018).

Virally derived TAT sequences can elicit immune responses and cause membrane toxicity at higher doses (Saar et al., 2005). However, species-specific absorption variability has prevented human trials. In monolayer Caco-2 cells, CPP-insulin conjugates improved intestinal absorption 6–8-fold (Liang and Yang, 2005).

Alternative oligoarginine cationic CPP designs also exist. Zhang et al. (2015) described R6EW, a modified R8 peptide containing six arginine residues, as well as glutamate and tryptophan. In their study, stable complexes between R6EW and insulin through hydrophobic π–stacking and guanidinium–carboxylate interactions. These complexes protect insulin from enzymatic degradation by trypsin and improve its transport across Caco-2 monolayers (Zhang et al., 2015).

SAR6EW, a stearylated derivative of R6EW, increases hydrophobicity and membrane affinity. This enhances non-covalent interactions with insulin and improves transepithelial transport. In diabetic rats, SAR6EW–insulin complexes exhibit low acute toxicity and prolonged hypoglycemic effects. Moreover, their relative bioavailability is higher than that of R8–insulin (Nielsen et al., 2014; Zhang et al., 2015). However, absolute oral bioavailability remains lower than that of subcutaneously administered insulin showing that mucosal barriers remain a major challenge (Nielsen et al., 2014).

By combining functionally distinct peptide domains, modular synthetic CPPs, such as the GET system, enable non-invasive insulin transport. GET’s original design (P21-8R) binds cell-surface glycosaminoglycans and initiates endocytosis by combining a heparan sulfate-binding domain (P21) with an octaarginine (8R) tail (Dixon et al., 2016). To facilitate endosomal escape of the cargo, PLR (P21-LK15-8R) adds an amphipathic LK15 segment between the P21 and 8R domains (Osman et al., 2018; Rehmani et al., 2023; Wong et al., 2024).

GET-insulin nanocomplexes increase intestinal and nasal transepithelial transport by over 22-fold (Rehmani et al., 2023). While meeting preclinical safety standards, these nanocomplexes corrected glucose levels in diabetic animals after oral or intranasal delivery (Rehmani et al., 2023). *In vitro*, the PLR variation increased insulin transport across RPMI 2650 nasal epithelial barriers (Wong et al., 2024). These conjugates have low absolute systemic absorption. As of early 2026, no GET-insulin formulations have entered clinical trials yet due to scaling challenges, regulatory concerns, and the need for further toxicity data.

In 2003, Dom et al. reported that penetratin, a 16-mer Antennapedia homeodomain-derived CPP, facilitates cellular uptake via a two-step translocation process (Dom et al., 2003).

Analogs of D-penetratin form stable, non-covalent complexes with insulin that withstand protein degradation (Birch et al., 2023; Diedrichsen et al., 2023). In Caco-2 monolayers, D-penetratin-insulin complexes demonstrate better permeability than insulin (Birch et al., 2023). Penetrating insulin conjugates increase insulin exposure in rodents compared to insulin alone (Khafagy et al., 2015). Ileal administration is especially efficacious (Khafagy et al., 2015). Stability and proteolysis reduction are improved by D-isomer amino acid CPP conjugates. Higher concentrations cause cytotoxicity and variable bioavailability, limiting clinical use (Khafagy et al., 2015). *In vitro*, branched penetratin dimers and trimers, as well as penetramax dimers and trimers, increase insulin delivery (Diedrichsen et al., 2025). Insulin-branched penetratin and penetramax dimers and trimers have lower absolute bioavailability, and there are currently no clinical trials using these oligomers.

In addition to insulin, CPP-based strategies have been applied to improve oral delivery of GLP-1RAs (Khafagy el et al., 2009). Exendin-4 and liraglutide conjugated to penetratin or other CPPs showed improved *in vivo* and *in vitro* absorption compared to unconjugated GLP-1RAs (Khafagy el et al., 2009, Uhl et al., 2020; Shams et al., 2026), with penetratin increasing nasal bioavailability of GLP-1 from 5% to 15.9% and of exendin-4 from 1.8% to 7.7% in rats (Khafagy el et al., 2009). Semaglutide (Rybelsus), the only approved oral GLP-1RA, relies on an SNAC absorption enhancer rather than a CPP, and no human CPP–GLP-1RA trials have been reported to date (Solis-Herrera et al., 2024; Wang et al., 2025). Metformin and SGLT2 inhibitors do not require CPP-mediated delivery augmentation: metformin achieves adequate oral bioavailability (39%–71%) via endogenous intestinal transporters (Graham et al., 2011), while SGLT2 inhibitors such as dapagliflozin and empagliflozin are small molecules with inherently high oral bioavailability that readily traverse cell membranes without assistance (Scheen, 2014; DeMarsilis et al., 2022). Similarly, PPARγ agonists pioglitazone (∼83% bioavailability) and rosiglitazone (∼99%) are nuclear receptor ligands that do not require CPP-assisted membrane crossing (Cox et al., 2000; Hanefeld, 2001). CPP-mediated delivery is therefore most rationally pursued for macromolecular cargoes -insulin, GLP-1RAs, and nucleic acid therapeutics -that face genuine membrane and mucosal barriers (Rehmani and Dixon, 2018).

Importantly, clinically successful metabolic delivery systems currently rely predominantly on receptor-mediated or absorption-enhancing strategies rather than CPP-mediated transport. Oral semaglutide uses the absorption enhancer SNAC rather than CPP-based transport, highlighting that clinically realistic, non-invasive delivery approaches may require simpler, more scalable formulations than current CPP-conjugated systems.

### 2.4 Challenges in clinical implementation

Cell-penetrating peptides are promising T2D drug delivery vehicles. They promote epithelial transcytosis, thereby improving the gastrointestinal absorption of therapeutics such as insulin, GLP-1RAs, and small interfering RNA (siRNA) (Rehmani and Dixon, 2018). This approach also offers a lower toxicity profile compared to viral vectors, although nanoparticle formulation can affect the outcome. CPP-driven uptake gain is dependent on the experimental model. In intestinal loop preparations, CPPs have been shown to significantly enhance insulin absorption (Kamei et al., 2008). For example, Liang and Yang (2005) reported 6–8-fold increases in Caco-2 monolayer CPP-insulin complexes (Liang and Yang, 2005).

Oligoarginine- and TAT-insulin conjugates release 27- and 19-fold more insulin, respectively, compared with unconjugated insulin in lung preparations (Patel et al., 2009). GET–insulin nanocomplexes show an approximately 22-fold higher transport across intestinal epithelial models (Rehmani et al., 2023). However, different experimental settings show differing outcomes.

Despite encouraging results, progress toward clinical translation is hampered by inefficient endosomal escape, limited tissue selectivity, and challenges in scalable manufacturing (Korivi et al., 2021; Nandhini et al., 2023). CPPs and therapeutic compounds must also overcome poor intestinal mucosal transepithelial permeability and rapid degradation by trypsin and chymotrypsin (Kristensen et al., 2015; Rehmani and Dixon, 2018) thereby increasing manufacturing complexity (Nandhini et al., 2023). Endosomal escape is challenging, but encapsulating cargos in protective nanoparticles or chemically altering CPPs can improve cytoplasmic delivery (Erazo-Oliveras et al., 2012)*.* Most CPP cargo is endocytosed (Richard et al., 2003) and delivered to lysosomes (Erazo-Oliveras et al., 2012). Histidine-rich, pH-sensitive CPP designs offer a potential solution to endosomal entrapment (Ruseska and Zimmer, 2020).

Many CPPs accumulate in tissues via non-selective electrostatic interactions with heparan sulfate proteoglycans (Tyagi et al., 2001), which may cause toxicity at therapeutic dosages (Saar et al., 2005). Membrane rupture and cytotoxicity were observed at values above 10 µM, supporting direct translocation (Saar et al., 2005). CPPs are mostly endocytosed at physiological concentrations, although cytosolic access is minimal (Ruseska and Zimmer, 2020). TAT has a 2% cytosolic delivery efficiency, but improved cyclic CPPs can achieve up to 120% (Buyanova and Pei, 2022). A significant fraction of absorbed TAT is retained within endosomes (Jiao et al., 2024), requiring modification of the CPP sequence and stoichiometry to attain sufficient cytosolic concentrations (Jiao et al., 2024). Cationic CPPs, when administered continuously, can, in theory, activate Toll-like receptors or the complement system in patients with T2D. This occurs despite their generally lower immunogenicity relative to viral vectors (Korivi et al., 2021).

A CPP-based oral insulin delivery system, when combined with the only approved oral peptide formulation for T2D, semaglutide (Rybelsus), demonstrates 1% absolute oral bioavailability using the SNAC absorption enhancer (Aroda and Blonde, 2022; Solis-Herrera et al., 2024). Compared with other formulations, GET insulin nanocomplexes have been shown to increase transport across epithelial models by 22-fold. The overall systemic absorption of GET insulin nanocomplexes remains low, and as of early 2026, no clinical trial data are available. Furthermore, although branched penetratin and penetramax dimers have demonstrated improved *in vitro* intestinal insulin delivery, neither has entered clinical development (Diedrichsen et al., 2025).

The translational requirements for T2D differ substantially from those in oncology or infectious disease, where most CPP and PNA data originate. T2D requires lifelong treatment, affects hundreds of millions of patients, so dosing frequency, pharmacokinetics, and cost must meet strict requirements. So far, all available research on CPP–insulin formulations relies on single-dose testing only. There is no published evidence yet describing immunogenicity upon repeated use or chronic toxicity outcomes in the T2D model (Korivi et al., 2021). Unmodified PNAs have a plasma half-life of approximately 17 min in mice due to rapid renal clearance, which is incompatible with practical dosing intervals for a chronic disease (McMahon et al., 2002). Both PEGylation and lipid nanoparticle formulations are used widely to prolong circulation time. (Ge et al., 2020). Detailed pharmacokinetic profiles in metabolic disease models are still insufficiently characterized. A relevant benchmark is inclisiran, a GalNAc-siRNA dosed twice yearly for the treatment of hypercholesterolemia. For PNA-based treatments to be given as often as existing therapies, their formulation will need to be greatly refined, and there is still no evidence that this is feasible for metabolic targets (Raal et al., 2020). PNA solid-phase synthesis is more complex and costly than standard oligonucleotide chemistry. Developing GalNAc-conjugated technologies from early design to industrial-scale production took about 15 years of dedicated process engineering and refinement (Springer and Dowdy, 2018; Debacker et al., 2020). Antibody-based delivery systems are costly for administration. This may be acceptable for short-term or targeted cancer therapy, but it is much harder to justify for chronic metabolic disorders. Cost-effectiveness modeling and scalable manufacturing should therefore be addressed in parallel with pharmacological optimization.

## 3 PNAs as therapeutic agents for gene modulation in T2D

Synthetic nucleic acid analogs, PNAs, bind complementary sequences with remarkable stability and sequence accuracy (Gupta et al., 2017; Singh and Monga, 2023). PNAs are nucleic acid analogs capable of binding to nanocarriers or CPPs, facilitating sequence-specific gene alteration (Gupta et al., 2017; Singh and Monga, 2023)**.** The charge-neutral pseudopeptide backbone of PNAs allows high-affinity, nuclease-resistant silencing of DNA and RNA targets. This is unlike CPPs, which cross the membrane and mucosal barriers (Gambari, 2001; Saarbach et al., 2019). PNA-mediated gene silencing has been demonstrated in oncology and infectious diseases (Economos et al., 2020; Wojciechowska et al., 2020; Dhuri et al., 2021; Shai et al., 2022; D'Aniello et al., 2026) but has yet to be applied to metabolic disease targets. Evidence for PNA efficacy in T2D models, therefore, remains indirect. In principle, PNAs could be designed to lower the expression of β-cell stress, diabetic-complication, or insulin-resistance genes such as PTP1B and PTEN; targets that have been successfully modulated with ASOs (Butler et al., 2002; Zinker et al., 2002).

To date, no trials have validated PNA-based techniques in diabetic models. However, CPP-PNA conjugates have shown robust cellular uptake and sequence-specific target repression across diverse therapeutic settings (Bendifallah et al., 2006; Shiraishi and Nielsen, 2011), supporting the use of CPP-based delivery and PNA-mediated gene modification. Despite minimal metabolic model testing, flexible, tailored structures that integrate peptide-based delivery with gene-modulating compounds could be developed for diabetes.

The pseudopeptide backbone N-(2-aminoethyl)-glycine, which is charge neutral, replaces the standard negatively charged sugar-phosphate backbone of RNA or DNA. Natural or modified nucleobases attach to this backbone (Saarbach et al., 2019; Singh and Monga, 2023). This modification confers stronger hybridization affinity to PNAs. Consequently, their melting temperatures are ∼1 °C higher per base pair than those of comparable standard DNA duplexes (Fouz and Appella, 2020) due to the absence of backbone electrostatic repulsion and high resistance to nucleases and proteases (Demidov et al., 1994). PNAs also do not rely on Ribonuclease H (RNase H) or RNA-Induced Silencing Complex (RISC) complexes for gene silencing, unlike small interfering RNAs (siRNAs) and many phosphorothioate ASOs (Bonham et al., 1995). Rather, they directly block mRNA translation or DNA transcription via steric binding (Bonham et al., 1995), thereby reducing cleavage-dependent off-target effects associated with other conventional oligonucleotide strategies.

PNAs' physicochemical features, therefore, make them appropriate antisense and antigene agents for sequence-specific manipulation of T2D pathways (Nielsen and Haaima, 1997; Gambari, 2001).

### 3.1 PNAs: structural and functional advantages

**FIGURE 4:**
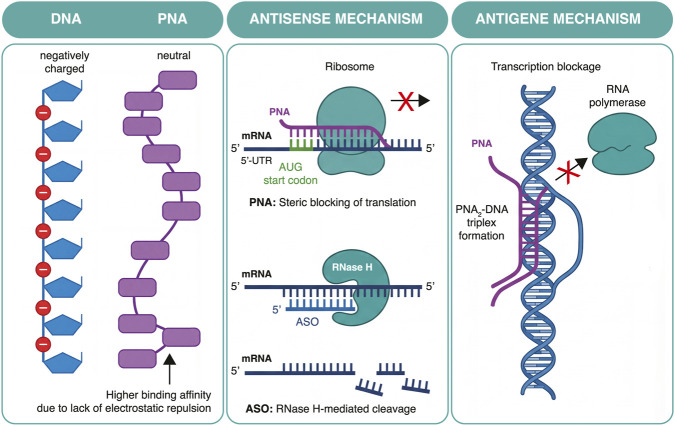
PNA backbone structure and gene silencing mechanisms DNA has a charged sugar–phosphate backbone, whereas PNA has a neutral N-(2-aminoethyl) glycine backbone. In antisense mechanisms, PNAs bind to the 5′-UTR/AUG of the PTP1B mRNA, hindering ribosomal scanning by steric block. This is unlike antisense oligonucleotides (ASOs), which rely on RNase-H-dependent cleavage. Antigene strategies: PNA strands invade DNA to form PNA_2_–DNA triplexes, which block RNA polymerase activity.

By reducing electrostatic repulsion between strands, PNAs' neutral backbone forms stable duplexes and PNA_2_-DNA triplexes that suit antigen methods (Figure 4) (Egholm et al., 1993; He et al., 2009). In enzyme-rich environments where multiple RNA-based compounds degrade rapidly, PNA stability and activity can be enhanced by additional modifications (Demidov et al., 1994; Sahu et al., 2011; Gupta et al., 2017). Derivatives with engineered backbones, including γ-substituted PNAs, strengthen conformational preorganization and binding affinity by adopting a right-handed helix (Yeh et al., 2010). Duplex and triplex stability are further enhanced by gem-dimethyl and related sterically-constrained analogs (Ganesh et al., 2007; Gourishankar and Ganesh, 2012). Cellular PNA absorption can also be further improved by cationic γ-modifications, such as aminomethylene groups (Mitra and Ganesh, 2012).

### 3.2 PNAs: antisense and antigene mechanisms

By binding to target mRNAs, sterically blocking translation initiation or ribosomal progression, and thereby decreasing protein synthesis, PNAs exert antisense effects (Knudsen and Nielsen, 1996). The most effective PNAs for blocking this translation are those that form duplexes with the 5′-UTR or an AUG start codon (Knudsen and Nielsen, 1996; Doyle et al., 2001). Protein elongation within the coding area can be prevented by triplex-forming PNA constructs (Knudsen and Nielsen, 1996). When administered properly, steric-blocking PNA constructs can result in significant knockdown in preclinical animals compared with RNase H-dependent phosphorothioate ASOs, whose construct efficacy is affected by delivery method (Sazani et al., 2001).

Several studies have shown that PNAs can interact with double-stranded DNA in an antigenic form to create duplex or even triplex structures capable of blocking the binding of transcription factor and the progression of RNA polymerase (Nielsen et al., 1994; Pooga et al., 2001; He et al., 2009). These PNA-DNA complexes mediate sequence-specific transcriptional repression due to their high thermal stability (He et al., 2009). PTP1B, a target confirmed in phosphorothioate ASO investigations, is one of the T2D genes whose transcription or translation may be inhibited by PNAs (Zinker et al., 2002). PNAs may also confer pharmacokinetic advantages over less-resistant oligonucleotide chemistries (McMahon et al., 2002).

### 3.3 Applications of PNAs in diabetes and insulin-related pathways

Chronic inflammation, β-cell dysfunction, and deregulation of insulin transcription are all involved in the development of T2D (Halban et al., 2014; Kahn et al., 2014). Numerous molecular targets within these pathways have been validated by ASO-based approaches. By increasing insulin signaling in the liver and adipose tissue, PTEN knockdown by phosphorothioate ASOs has been shown to restore normal blood glucose levels in diabetic mice (Butler et al., 2002). Furthermore, ASOs targeting PTP1B increased insulin sensitivity and reduced hyperglycemia in ob/ob and db/db mice (Zinker et al., 2002). Despite the absence of PNAs targeting these genes, these findings provide a rationale for sequence-specific gene regulation in T2D.

Peptide nucleic acid have a plasma half-life of approximately 17 min in unmodified form (McMahon et al., 2002). They do not have the ability to recruit RNase H or RISC. They require stoichiometric target engagement. Their neutral backbone confers higher binding affinity, greater nuclease resistance, and an absence of Toll-like receptor 9 (TLR9)-mediated immunostimulation (Ray and Nordén, 2000; Upadhyay et al., 2008). These properties position PNAs as complementary platforms, with ASOs and siRNAs. PNAs are particularly useful in applications where high sequence specificity and a lack of immunogenicity are the main priorities.

The only direct application of PNAs in metabolic disease to date involves miR-33 PNA inhibitors. In animal models of chronic kidney disease, they reduce kidney fibrosis by restoring fatty acid oxidation (Price et al., 2019). According to one study, PNAs produce functional gene silencing (Doyle et al., 2001). Separately, *in vivo* studies have demonstrated distribution to metabolically relevant tissues, supporting their potential for target key physiological pathways (McMahon et al., 2002). PNAs could target genes validated by ASO platforms, including PTP1B, PTEN, and profibrotic microRNAs (miRNAs). Engineered PNAs hold greater enzymatic stability than ASOs, although experimental tests in metabolic models remain absent. Despite their attractive physicochemical profile, progress in PNA therapeutics is slower than for other ASO platforms, largely due to poor intrinsic cellular uptake, limited solubility, and rapid renal elimination (Gupta et al., 2017). Current PNA research centers on antiviral, antibacterial, and oncologic applications, with no PNA-based medicines yet tested clinically for metabolic disorders (Gupta et al., 2017). This gap reflects the biochemical potential of PNAs that has yet to be applied to T2D.

We propose that validation of PNA efficacy in type 2 diabetes models should follow a stepwise preclinical program. The pipeline may begin with *in vitro* validation of the relevant targets. The proposed work focuses on PNAs targeted to the 5'-UTR, or AUG start region of *PTP1B* and *PTEN* transcripts (Zinker et al., 2002; Clampit et al., 2003). Each sequence should be tested in three different configurations, such as unmodified naked PNA, conjugated to a cell-penetrating peptide, or linked to a GalNAc moiety. These should be tested in hepatocyte cell lines, including HepG2 and primary mouse hepatocytes, as well as in the adipocyte 3T3-L1 cell line. Target gene knockdown, together with downstream signaling proteins such as pAkt and pIRS-1, would help identify the carrier system with the best intracellular delivery (Gum et al., 2003). This would also indicate which system achieves the greatest cytosolic availability. The optimized construct would subsequently be tested in ob/ob or db/db diabetic mice. These are the same models used to validate phosphorothioate ASOs targeting PTP1B (Zinker et al., 2002). The primary endpoints would be hepatic PTP1B mRNA expression, fasting blood glucose, and insulin tolerance. Pharmacokinetic endpoints would measure tissue distribution in the liver, kidney, and pancreas, as well as plasma half-life. A matched phosphorothioate ASO control arm allows direct comparison between ASOs and PNAs. This design allows assessment not only of efficacy and duration of action, but also of off-target effects. To examine these differences at the molecular level, liver RNA-seq should be used to capture transcriptome-wide patterns (Michel et al., 2021). Finally, there is still a lack of long-term safety data for PNAs in metabolic disease models. For that reason, a dedicated safety assessment would be necessary before any translational progression. We suggest that this assessment include monitoring of multiple safety measures over the course of repeated treatment. These measures should investigate complement activation, the formation of anti-drug antibodies, structural changes in the kidneys, and the profile of circulating liver enzymes.

### 3.4 PNAs: delivery barriers and conjugation strategies

Despite their advantageous molecular properties, PNAs face several delivery challenges. Although their neutral, hydrophilic backbone provides high binding affinity and nuclease resistance, it also leads to poor cellular uptake, endosomal trapping, and restricted penetration into dense tissues crucial for T2D treatments, such as the pancreas and liver (Gupta et al., 2016; Gupta et al., 2017; Singh and Monga, 2023). Successful application of antisense or antigenic strategies in organs that are relevant to T2D requires PNA constructs that can effectively overcome intracellular and anatomical barriers in order to reach specific targets. Conjugation, or a combination thereof, is therefore required to effectively transport PNAs to relevant tissues (Shiraishi and Nielsen, 2006; Gupta et al., 2016; Gupta et al., 2017).

**FIGURE 5:**
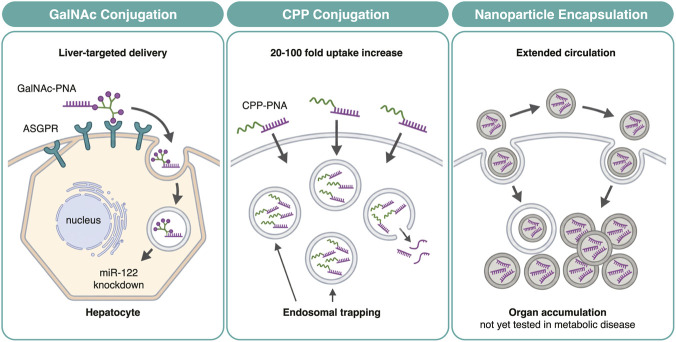
PNA delivery strategies: conjugation and nanocarrier approaches GalNAc–PNA conjugates bind ASGPR on hepatocytes. This interaction leads to their entry into the cells via receptor-mediated endocytosis. This process enables liver-directed delivery, for example, in targeting miR-122 knockdown. CPP–PNA conjugates (TAT, penetratin) dramatically increase cellular uptake by 20-100 times. The majority of the cargo remains trapped within endosomes, limiting the amount of cargo that reaches the cytosol. Encapsulating nanoparticles (LNPs, polymeric particles) helps them circulate longer and accumulate more in organs.

Three complementary PNA delivery methods are the focus of current research: (i) N-acetylgalactosamine (GalNAc) ligands that facilitate uptake into hepatocytes via the asialoglycoprotein receptor (ASGPR); (ii) CPPs to improve cellular penetration efficiency; and (iii) nanocarriers such as polymeric nanoparticles or lipid that protect circulating PNAs and enable suitable biodistribution (Figure 5) (Bendifallah et al., 2006; Gupta et al., 2016; Kumar et al., 2023).

Conjugates, such as ***GalNAc-PNA,*** have demonstrated proof of concept for liver-targeted PNA administration and ASGPR-dependent hepatocyte uptake (Kumar et al., 2023). To date, neither the glucagon receptor (GCGR) nor PTP1B has been targeted by a GalNAc-PNA combination. Similarly, no studies have assessed CPP-PNA constructs in adipocytes or other metabolism-related cell models. CPP-PNA constructs have not been tested in adipocytes or other metabolically relevant cell types.

In optimized systems, PNAs linked to CPPs such as penetratin or TAT show improved cellular uptake by 20-fold up to 2 orders of magnitude compared with unconjugated PNAs (Bendifallah et al., 2006; Koppelhus et al., 2008; Shiraishi and Nielsen, 2011). Most CPP-PNA conjugates cross the cellular membrane via endocytosis and remain largely sequestered in endosomes (Erazo-Oliveras et al., 2012). Only a small fraction of CPP-PNA conjugates reaches the nucleus or the cytosol, which usually requires auxiliary endosomolytic agents (Abes et al., 2006; Shiraishi and Nielsen, 2006). Several endocytic pathways have been reported for CPP-cargo systems, including caveolin- and clathrin-dependent endocytosis and macropinocytosis. These are influenced by cargo characteristics, conditions, and CPP sequence (Lundin et al., 2008; Swanson, 2008).

MiRNA suppression without CPP or nanoparticle conjugation is made possible by thyclotides (tetrahydrofuran-modified PNAs) and other backbone-engineered compounds that combine improved intrinsic cellular uptake with increased nucleic acid affinity (Clausse et al., 2022; Zheng et al., 2023). However, these findings were obtained in cancer cell models, and equivalent data in metabolic cell types are lacking. Rapid renal clearance is one of the pharmacokinetic constraints of PNAs (McMahon et al., 2002). Lipidation and nanoparticle encapsulation of these constructions can prolong circulation duration and enable organ-directed accumulation (Gupta et al., 2016; Menacho-Melgar et al., 2019; Sarkar and Murthy, 2026). In preclinical models, lipid nanoparticles originally developed for mRNA delivery are also efficient at delivering PNAs to pancreatic cells. Although no lipid nanoparticle (LNP)-PNA formulation has been evaluated in metabolic disease, these findings suggest a potential route for PNA delivery to this organ (Sarkar and Murthy, 2026). Although mucoadhesive polymer carriers show better oral and nasal absorption of peptide and insulin formulations (Khutoryanskiy, 2011), their use in PNA delivery has not yet been explored.

Despite their advantageous biophysical properties, PNAs still encounter several interconnected barriers on the path to clinical translation. Synthesizing PNAs is considerably more expensive than synthesizing ASOs or siRNAs.This is due to the use of protected monomers in solid-phase peptide chemistry. Unmodified PNAs are cleared rapidly by the kidneys. In mice, the plasma half-life is approximately 17 min (McMahon et al., 2002). By contrast, GalNAc–siRNA conjugates achieve hepatic half-lives of days to weeks (Springer and Dowdy, 2018). The neutral, hydrophilic pseudopeptide backbone of PNAs, which provides nuclease resistance and binding affinity, simultaneously prevents passive membrane crossing (Ray and Nordén, 2000). According to experimental data, only 1%–2% of internalized nucleic acid agents successfully escape endosomes to reach their site of action in the cytosol or nucleus (Allen and Yokota, 2024). CPP conjugation improves cellular uptake by 20-fold to two orders of magnitude. However, most CPP–PNA conjugates remain trapped in endosomes. They require auxiliary endosomolytic agents for cytosolic delivery (Abes et al., 2006; Shiraishi and Nielsen, 2006). Tissue targeting is still not well developed. For example, GalNAc–PNA conjugates have only shown proof-of-concept miR-122 knockdown in hepatocytes (Kumar et al., 2023). No PNA constructs currently exist that specifically target metabolic proteins such as PTP1B, PTEN, or GCGR. In contrast to RNase-H-dependent ASOs or RISC-dependent siRNAs, PNAs rely on stoichiometric steric blocking. Because of this, they must reach much higher intracellular concentrations to work effectively, but delivery remains a major challenge. Overcoming these barriers will require rigorous preclinical evaluation. This evaluation should compare naked PNA, CPP–PNA, and GalNAc–PNA scaffolds in hepatocyte and adipocyte cell lines. Next, *in vivo* testing should be performed in ob/ob or db/db diabetic mice (Zinker et al., 2002). These studies should include direct benchmarking against a matched phosphorothioate ASO. A dedicated safety assessment is also needed. We suggest assessing complement activation, anti-drug antibody responses, plus kidney and liver function, especially after the compound has been dosed repeatedly.

#### 3.4.1 Pharmacological and safety profiles of PNAs

According to Nandhini et al. (2023) and Singh and Monga (2023), PNA synthesis is more comparable to peptide chemistry than DNA chemistry (Nandhini et al., 2023; Singh and Monga, 2023). The pseudopeptide backbone and chosen nucleobase sequence are assembled by PNAs using solid-phase peptide synthesis (SPPS) (Nandhini et al., 2023; Singh and Monga, 2023). By forming right-handed helices, chiral γ-PNAs enable high-affinity binding to complementary strands (Dragulescu-Andrasi et al., 2006; Yeh et al., 2010) and may reduce the entropic cost of hybridization. Thyclotide pseudopeptide backbones stabilize duplexes. These backbones also enhance cellular uptake and miRNA inhibition, particularly when constructs are well-designed (Clausse et al., 2022; Zheng et al., 2023). PNAs are commonly modified by attaching short polyethylene glycol (PEG) chains, CPPs, receptor-targeting ligands, or GalNAc (Gupta et al., 2016; Kumar et al., 2023). Due to their high sequence complementarity, off-target hybridization is reduced, as single-base mismatches decrease duplex stability by 15–20° C, resulting in high target specificity (Igloi, 1998; Ratilainen et al., 2000).

Since no PNA has yet been tested for T2D, PNA-based metabolic disease medicines have not been thoroughly evaluated for safety. PNA safety profiles cannot be extrapolated from the tolerability records of chemically distinct phosphorothioate ASOs, and unmodified PNAs have a low immunogenicity profile (Upadhyay et al., 2008). However naked PNA has been shown to be immunologically inactive in mice (Upadhyay et al., 2008) and to lack the cytosine–phosphate–guanine (CpG) dinucleotide motifs and phosphodiester backbone recognized by classic TLR9 (Hemmi et al., 2000).

The CPP-PNA conjugates' peptide component can generate modest immunological responses (Upadhyay et al., 2008), which should be considered for administration techniques. According to McMahon et al. (2002), unmodified PNAs have a plasma half-life of 17 min in mice and are rapidly cleared by the kidneys (McMahon et al., 2002). Conjugation or PEGylation to higher-weight molecular carriers can prolong the circulation time of PNA, as seen with comparable peptide therapies (Veronese and Mero, 2008). However, there is limited evidence of PNA-specific PEGylation pharmacokinetic investigations to date.

### 3.5 Clinical translation and future directions

Peptide nucleic acid treatments are behind licensed peptide- and RNA-based oligonucleotide medicines, and no PNA has been clinically tested for any metabolic indication to date. Machine learning-guided prediction of synthesis efficiency and automated synthesis may help overcome scalability barriers in manufacturing (Li and Zhang, 2022). The physicochemical characteristics of γ-PNA and alterations to the thyclotide backbone have been reported to improve binding affinity, structural preorganization, and cellular uptake (Dragulescu-Andrasi et al., 2006; Clausse et al., 2022). Once delivery and absorption challenges are addressed, PNA therapies could follow antisense oligonucleotides and GalNAc-conjugated siRNAs in metabolic disorders, demonstrating that the sequence-specific gene regulation can effectively target genes expressed in the liver (Springer and Dowdy, 2018; Debacker et al., 2020).

Peptide nucleic acid may be integrated with receptor-targeted delivery systems in order to modulate expression of genes in specific tissues. However, this has not yet been tested in models of metabolic diseases. Mechanistic evidence comes from ASO studies in diabetic mice that target PTEN and PTP1B (Butler et al., 2002; Zinker et al., 2002), along with the established *in vivo* activity of anti-miR-33 PNA in kidney disease (Price et al., 2019). This indicates that PNA-based strategies targeting metabolic pathways show promise. Early-stage clinical trials will depend on advanced delivery technologies (Saarbach et al., 2019) and manufacturing-compatible processes (Nandhini et al., 2023).

## 4 Antibody/Receptor-mediated delivery

Antibody receptor-mediated delivery represents a novel approach to targeted therapy. It is based on the use of antibodies or ligands for specific receptors to direct therapeutic agents to certain cells or tissues selectively.

The modular structure of immunoglobulins can be used to design and create “custom” drugs, whose pharmacological properties can be used for specific therapeutic needs. Due to their defined functional domains, antibodies can be selectively modified or removed to create molecules with specific characteristics, such as improved binding specificity, enhanced tissue penetration, and greater stability (Nelson, 2010; Fernandes, 2018).

Monoclonal antibodies (mAbs) have long been the preferred technique in biomedicine, as they exhibit excellent specificity, can be engineered to bind large quantities, and can be engineered to bind to almost any antigen. Additionally, their production enables relatively good standardization. On the other hand, the development of hybridoma technology by Kohler and Milstein in 1975 opened the possibility to generate mAbs with precise specificity consistently. Hybridoma cells can be used to provide smaller functional antibody fragments, such as the fragment antigen-binding (Fab) or single-chain variable fragment (scFv), which are created by extracting and combining only the variable domains of antibodies (Ahmad et al., 2012).

Single-chain variable fragment is a small fragment of a recombinant antibody, and it consists of heavy and light chain variable domains, which are linked by a flexible peptide linker into a single polypeptide chain. They can specifically bind antigens. Their structure enables additional modifications leading to improved affinity and specificity (Ahmad et al., 2012).

Fragment antigen-binding is a larger fragment and represents the oldest class of therapeutic monoclonal antibody fragments. They are derived from intact immunoglobulins, usually Immunoglobulin G (IgG) or Immunoglobulin M (IgM). Fab contains the complete light chain, the variable regions of the heavy chain, and their constant regions (CH1 domain and CL). They are connected by disulfide bonds, which allow them to bind antigens, even though they are smaller than a whole antibody. Fab lacks the Fc region, which is responsible for effector functions (Ahmad et al., 2012).

Overexpression of specific receptors/antigens allows selective targeting of tumor cells. Antibody-mediated drug delivery has demonstrated substantial clinical success across diverse tumor types, particularly through the development of antibody–drug conjugates (ADCs) (Metrangolo and Engelholm, 2024). These systems, which cause minimal damage to healthy tissue, are composed of mAbs chemically linked to a cytotoxic agent, allowing direct delivery of the drug to the tumor cell. So far, the Food and Drug Administration (FDA) has approved 14 ADCs, while clinical studies are ongoing to assess others (Sanjanwala et al., 2025).

On the other hand, noncovalent antibody-drug complexes (ADCx) represent a still under-researched approach in drug delivery. Although there are some promising early-stage studies, more detailed investigations and refinement of these systems are necessary to understand their full potential. ADCx may be particularly appropriate for delivering drugs that cannot be delivered using classical ADC systems, for instance, due to the absence of suitable functional groups for binding linkers or an antibody. Overall, the use of ADCx could be an encouraging targeted therapy approach (Sanjanwala et al., 2025).

### 4.1 Therapy for beta-cell regeneration and targeted insulin delivery

Therapeutic agents can bind to specific receptors on the cell surface (Alavi and Ebrahimi Shahmabadi, 2021). This mechanism enables the selective delivery of drugs directly to target cells or tissues, thereby increasing therapy effectiveness and reducing unwanted systemic effects. In conditions such as diabetes, the development of techniques that use the antibody/receptor-mediated delivery system combined with nanotechnology can create new opportunities for more effective and safer treatment, particularly in patients with impaired β-cell function (Collins and Farnsworth, 2025). Pancreatic β cells are key targets for therapy in type 1 and T2D diabetes (Knerr et al., 2021).

Important candidates for clinical application are antibody-coated nanoparticles. So far, some antibody-based targeting methods are already in use for imaging β-cells, many are yet to be tested in human models, including ectonucleoside triphosphate diphosphohydrolase-3 (ENTPD3) and transmembrane protein 27 (TMEM27), both of which were expressed on human pancreatic β-cells (Collins and Farnsworth, 2025). In pancreatic β cells, the incretin peptide glucagon-like peptide-1 receptor (GLP-1R) could represent an important target given its high expression. As a result, ligands based on glucagon-like peptide-1 (GLP-1) could be used to deliver drug conjugates toward the cells, leading to more precise and efficient delivery (Alavi and Ebrahimi Shahmabadi, 2021).

**FIGURE 6:**
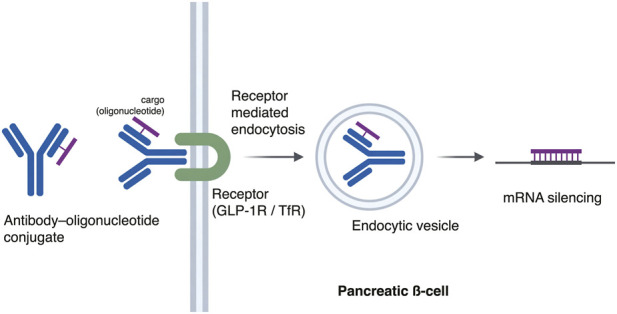
Schematic representation of the delivery and intracellular activity of an AOC. The antibody–oligonucleotide (AOC) conjugate consists of a monoclonal antibody linked to a therapeutic oligonucleotide cargo. The antibody part targets specific cell-surface receptors. The antibody moiety targets specific cell-surface receptors such as GLP-1R on pancreatic β-cells and TfR. After receptor binding, the AOC enters an endocytic vesicle, and the oligonucleotide cargo is then released, triggering mRNA silencing.

Aptamers could also be used for targeted binding to molecules. Aptamers, which are oligonucleotide sequences, can also be conjugated to various nanoparticles to achieve direct targeting (Collins and Farnsworth, 2025). Recent studies have shown that conjugating (ASOs to GLP-1 receptor agonists enables selective delivery and effective uptake in these cells, highlighting a promising strategy for targeting this important cell population (Knerr et al., 2021). The mechanism of action is based on the high specificity of the interaction between the antibody (or ligand) and the corresponding receptor. After binding to a cell–surface receptor, the ligand–receptor complex can be internalized via receptor-mediated endocytosis (Figure 6). This process includes cell membrane invagination and the formation of endosomes, allowing the therapeutic agent to enter the cell interior. After that, the therapeutic agent can be delivered in the cytoplasm or transferred to intracellular organelles, where it can exert its full effect (Sung et al., 2025). Insulin can also be modified or conjugated to molethe neonatal Fc receptor (FcRn). The FcRn enhances its absorption and its transport across cells via receptor-mediated endocytosis (Aghakhani et al., 2025).

### 4.2 Advantages and challenges

One of the main benefits of antibody/receptor-mediated delivery systems is their capacity to target cells or tissues. Therapeutic agents can be recognized and bind to specific receptors on cell surfaces (Sanjanwala et al., 2025). In this way, the impact on healthy tissues is significantly reduced, thereby reducing side effects. In addition, this approach increases therapeutic efficiency by allowing a higher drug concentration to accumulate in target cells, leading to a better therapeutic response and a lower required dose. Smaller functional antibodies, such as scFv and Fab, can penetrate tissues more quickly and deeply. Also, due to their smaller size, they may bind to epitopes that are inaccessible to full-sized mAbs (Ahmad et al., 2012).

However, it is important to emphasize that there are several limitations to the use of traditional mAbs. Their therapeutic application is restricted due to an immunological reaction in humans known as HAMA (human anti-mouse antibody), since they are produced in mice. Their production is also sophisticated and difficult, expensive, and time-consuming (Ahmad et al., 2012). Fragments also have have shorter circulation half-lives in humans; they can be less stable than full-size mAbs and are more prone to forming undesirable aggregates. Also, unlike full-length mAbs, Fabs are unable to trigger effector functions since they lack the Fc region (Nelson, 2010). An additional problem is limited penetration into solid tissues, where large molecules such as antibodies have difficulty traversing the dense extracellular matrix, thereby reducing the effectiveness of therapy in certain conditions. Use of ADCs in cancer patients during treatment has been shown to lead to resistance (Li et al., 2025). On the other hand, ADCs' value in metabolic disease is still unclear. Applying similar delivery concepts to chronic metabolic diseases such as T2D is challenging. Several key receptors are important targets in T2D research. These include GLP-1R, the insulin receptor, TMEM27, and ENTPD3 on β-cells (Vats et al., 2012; Ämmälä et al., 2018; Saunders et al., 2019; Knerr et al., 2021), as well as ASGPR on hepatocytes. However, these targets are either broadly distributed across tissues or expressed at low densities on the intended cell populations. This makes selective targeting more difficult. The kind of cell-specific delivery that supports ADC success in oncology is more challenging in T2D. Binding nucleic acids to a chosen receptor can help deliver the material more selectively to the target. For example, GLP-1R-conjugated antisense oligonucleotides were delivered to pancreatic β-cells *in vivo* and reduced target gene expression in islets, with little or no effect in the liver or other tissues (Ämmälä et al., 2018). Similarly, hepatocyte-targeting with triantennary GalNAc improves antisense potency by approximately 6–10-fold. This effect is mediated by high-affinity binding to ASGPR. Also, multivalent GalNAc–siRNA conjugates produce robust RNAi-mediated silencing in hepatocytes *in vivo* (Nair et al., 2014; Prakash et al., 2014). Beyond approaches targeting the liver or β-cells more broadly, targeting TMEM27 has emerged as a way to achieve greater, more selective uptake in β-cells (Vats et al., 2012). ENTPD3-directed antibodies promote selective uptake by β-cells. They also support *in vivo* detection in human islet graft (Saunders et al., 2019). Unlike cancer, which requires a limited treatment course, T2D demands chronic, lifelong dosing. This raises additional concerns about cumulative Fc-mediated effector function risks, including complement activation and antibody-dependent cellular cytotoxicity. It also raises concerns about long-term immunogenicity. Meanwhile, the high manufacturing costs of full-length monoclonal antibodies are a major challenge for large-scale use in metabolic disease. Engineered formats such as AOCs have shown effective preclinical results, but no antibody-based delivery system has yet been approved for T2D.

Antibody receptor-mediated drug delivery is a promising approach in oncology and targeted gene therapy. A combination of antibody specificity and targeted transport of therapeutic agents could lead to the development of personalized therapeutic strategies that significantly improve clinical outcomes.

There are multiple barriers that limit the efficacy of antibody-targeted delivery systems for metabolic diseases.One primary concern is that these antibody-targeted delivery systems typically require multiple doses (often daily) for a long period of time; therefore, there are increased concerns regarding immunogenicity, safety, and off target effects. Also, full-length IgG antibodies have poor tissue penetration and can elicit Fc-mediated immune responses; in addition, many targets on β-cells, such as GLP-1R, TMEM27, and ENTPD3, are non-exclusive to β-cells and are expressed at relatively low levels, leading to very narrow therapeutic windows (Vats et al., 2012; Park et al., 2016; Collins and Farnsworth, 2025).

A comparison of CPP, PNA, antibody-based, and ASO/siRNA delivery platforms for metabolic disease is presented in Table 2.

**TABLE 2:** Comparison of CPP, PNA, antibody-based, and ASO/siRNA delivery platforms in metabolic disease.

Delivery platform	CPPs	PNAs	Antibody/Receptor-mediated	ASOs/siRNA
Delivery efficiency (*in vitro*)	6 to 27-fold increase ( Liang and Yang, 2005; Patel et al., 2009 )	20 to 100-fold with CPP conjugation ( Bendifallah et al., 2006; Shiraishi and Nielsen, 2011 )	High; receptor-mediated uptake ( Ämmälä et al., 2018; Knerr et al., 2021 )	High, depends on chemistry and delivery system, GalNAc, LNPs (Springer and Dowdy, 2018)
Plasma half-life	Minutes to hours (Ruseska and Zimmer, 2020)	∼17 min unmodified; longer with conjugation (McMahon et al., 2002)	Hours to weeks, depending on format (Nelson, 2010);	Hours to days with modification (Springer and Dowdy, 2018)
Target specificity	Moderate (Ruseska and Zimmer, 2020)	Very high ( Nielsen et al., 1994; Ratilainen et al., 2000 );	Very higha (Ämmälä et al., 2018)	High; some off-target effects
Tissue selectivity	Low (Korivi et al., 2021)	Low unless conjugated ( Gupta et al., 2017; Kumar et al., 2023 );	High; receptor dependent (Knerr et al., 2021)	Moderate to high (Springer and Dowdy, 2018)
Immunogenicity	Low–moderate (La-Beck et al., 2020);	Low (Hemmi et al., 2000);	Moderate–high; HAMA risk (Ahmad et al., 2012)	Low–moderate (Springer and Dowdy, 2018)
Manufacturing complexity	Moderate ( Kristensen et al., 2016; Nandhini et al., 2023 );	Moderate to high (Nandhini et al., 2023)	High ( Nelson, 2010; Ahmad et al., 2012 )	Moderate (Debacker et al., 2020)
Clinical status	Preclinical only ( Xie et al., 2020; Wang et al., 2025 )	No clinical trials (Gupta et al., 2017)	Preclinical data only (Ämmälä et al., 2018)	Multiple approved therapies ( Springer and Dowdy, 2018; Debacker et al., 2020 )

### 4.3 Combination strategies: integrating CPP-, PNAs-, and receptor-mediated delivery

**FIGURE 7:**
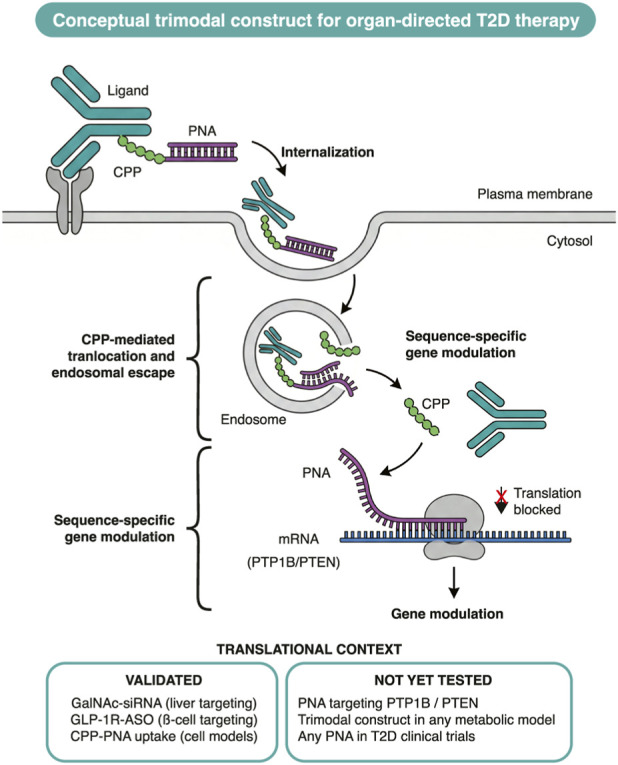
Proposed trimodal construct for organ-directed T2D gene silencing and translational status of its components The therapeutic construct comprises a targeting Ligand, a Cell-Penetrating Peptide (CPP), and a Peptide Nucleic Acid (PNA). This delivery mechanism starts with receptor-mediated internalization, then relies on CPP-mediated endosomal escape. Once in the cytosol, the PNA binds to target mRNA (such as PTP1B/PTEN) via sequence-specific recognition, effectively blocking translation and modulating gene expression. The Translational Context summarises currently validated targeting methods that have not yet been tested.

A multimodal strategy for T2D can be envisioned. This strategy would involve CPPs to transport molecules across cell membranes. Also, PNAs would act as stable gene-modulating cargo, while antibodies or endogenous ligands would ensure tissue-specific localization (Figure 7). In this way, CPPs provide cell entry, PNAs mediate sequence-specific effects, and antibodies or ligands restrict activity to selected tissues. To date, no testing of a trimodal CPP-PNA-antibody construct has been conducted. However, this strategy is supported by analogous multimodal structures for other oligonucleotide classes, and each component has been validated independently (Jiao et al., 2024). To our knowledge, there are no studies that have evaluated a trimodal construct composed of a cell-penetrating peptide (CPP), a peptide nucleic acid (PNA), and an antibody. This applies to all types of biological and preclinical model systems. This strategy is supported only by studies of its individual components and by analogous multimodal frameworks for other oligonucleotide classes (Tietz and Cortezon-Tamarit, 2022; Jiao et al., 2024; Li et al., 2025). Four related issues still need to be resolved before practical implementation.

Recent progress in cell-penetrating peptide design and multifunctional delivery systems provides strong proof of concept for these approaches.

In this context, Tietz (2022) created a trimeric CPP by linking three TAT(49–57) peptides to a rigid scaffold through click chemistry, followed by cyclization to form a tricyclic TAT construct. The trimer outperformed monomeric TAT at ten times the dose, achieving homogeneous cytosolic and nuclear delivery at just 1 µM. The construct linked to full-length IgG antibodies and Fab fragments, carrying functional antibodies to intracellular targets inside live cancer cells. The intended application is therapeutic modulation of undruggable intracellular cancer targets, such as oncoproteins and protein–protein interaction interfaces. However, all evidence to date is *in vitro* using cancer cell lines, with no data on metabolic or endocrine cell types (Tietz and Cortezon-Tamarit, 2022).

Receptor-mediated components: Antibody and ligand elements supply tissue specificity that CPPs lack. Ämmälä et al. (2018) developed a GLP-1/GLP-1R-targeted ASO that directed ASOs to pancreatic β-cells. Target gene knockdown occurred after ASO uptake (Ämmälä et al., 2018). *In vivo* knockdown of miR-122 by dilactobionic acid (diLBA) and triantennary galactosamine (tGalNAc)-PNA conjugates confirms GalNAc conjugation for hepatocyte-targeted PNA transport (Kumar et al., 2023). Receptor-targeting techniques could direct PNAs to liver metabolic targets such as PTP1B or GCGR, although no such constructs have been reported. Co-treatment with lysosomotropic agents or other endosomal-disruption strategies has enhanced cytoplasmic delivery of CPP-PNA conjugates following internalization (Shiraishi and Nielsen, 2006).

Cell-penetrating peptides modules: In targeted constructs, CPP domains can facilitate membrane passage and endosomal escape following receptor-mediated internalization and conjugate delivery to the target cell (Shiraishi and Nielsen, 2006). Amphipathic and Cationic CPPs, such as TAT, penetratin, and oligoarginine, have demonstrated increases in PNA uptake of 20-fold to 2 orders of magnitude in cell-based assays (Koppelhus et al., 2008; Shiraishi and Nielsen, 2011). However, naked CPP-PNA constructs show reduced tissue selectivity. Inserting CPPs between a targeting element and PNA cargo could address this issue, though such multilayer designs lack testing for metabolic targets. Delivery methods that are glucose-responsive, including glucose-sensitive polymers and phenylboronic acid, have emerged for insulin formulations (Matsumoto et al., 2012). These stimuli-responsive designs may conceivably be used for CPP-PNA platforms, although this option has yet to be investigated.

Peptide nucleic acids cargo: PNAs form a stable, sequence-specific gene-modulating platform. Metabolic targets such as PTP1B, PTEN, and GCGR have been successfully modulated in phosphorothioate ASO studies (Butler et al., 2002; Zinker et al., 2002; Sloop et al., 2004). However, no PNA has been directed against any of these genes to date. Studies in mice provided *in vivo* efficacy data for one metabolic PNA: the anti-miR-33 compound, which decreased kidney fibrosis (Price et al., 2019).

#### 4.3.1 From concept to clinical translation

For T2D, multimodal constructs incorporating CPPs, PNAs, and receptor targeting remain hypothetical. However, liver-directed GalNAc-siRNA delivery (Springer and Dowdy, 2018; Debacker et al., 2020) and β-cell GLP-1R-directed ASOs (Ämmälä et al., 2018) confirm that receptors can control gene activity in metabolic tissues. Receptors can therefore be used to guide gene modulation in metabolic tissues. In addition, dual- or multi-target pharmacological approaches such as GLP-1/amylin can outperform monotherapies. Constructs that integrate targeting, gene modulation, and efficient transport within a single conjugate are required (Jiao et al., 2024).

Proving basic feasibility is no longer the main obstacle; practical challenges now take center stage. Research must demonstrate that these sophisticated structures can improve metabolic outcomes at required doses, within demands, and at safety levels suitable for long-term diabetes therapy. Several limitations emerge. First, steric constraints arise because each functional domain -antibody Fab, CPP membrane-interaction surface, and PNA hybridization strand - requires spatial freedom that may be mutually restricted within a compact conjugate (Boisguérin et al., 2015; Tietz and Cortezon-Tamarit, 2022). Second, manufacturing is notably difficult. Each bond in a covalent trimodal conjugate should be formed using orthogonal chemistry. This makes the manufacturing process more challenging than for conventional oligonucleotide systems. This increases the possibility of incomplete conjugation and batch-to-batch variation (Ahmad et al., 2012; La-Beck et al., 2020; Moghimi et al., 2020). Third, immunogenicity represents concern as well. When a monoclonal antibody is combined with a CPP, it may trigger stronger immune responses, including HAMA responses and CPP-specific activation. Repeated dosing in long-term T2D treatment makes this risk higher (Ahmad et al., 2012; La-Beck et al., 2020). Fourth, moving through the regulatory pathway remains difficult. This is because no existing standards cover combined CPP–oligonucleotide–antibody therapies. For this reason, these constructs require comprehensive safety testing before clinical studies (Pasanen et al., 2017).

## 5 Conclusion and prospects for the future

Current T2D treatments successfully manage hyperglycemia, but they do not fully address underlying hepatic insulin resistance or β-cell loss. These inadequacies may be addressed by methods that enable the targeted delivery of medicines to tissues relevant to the condition. This review investigated three promising therapeutic delivery platforms: CPPs, PNAs, and antibody- or receptor-mediated targeting systems. Modulation of gene expression and medication delivery in diabetes was evaluated using available preclinical data. Each platform has distinct advantages but also presents considerable translational challenges.

Cell-penetrating peptides promote membrane and intracellular transfer of peptide cargos through direct translocation and endocytosis. However, oral bioavailability remains low in preclinical models. PNAs provide a nuclease-resistant system for sequence-specific gene knockdown or silencing and could potentially target metabolic regulators such as PTP1B and PTEN. However, most data supporting the efficacy of these pathways originates from phosphorothioate ASO research rather than PNA-based investigations in models of T2D.

Conceptual approaches that combine CPP-mediated intracellular access, PNA-based gene regulation, and receptor-guided targeting are mostly theoretical. No such systems have yet received regulatory approval for T2D therapy to date.

Several translational barriers remain for therapeutic delivery platforms for T2D. These include poor bioavailability of CPP constructs, endosomal trapping of CPP-PNA constructs, and potential immunogenicity of peptide-based carriers. Manufacturing costs are also high. The majority of data are limited to rodent models and *in vitro* cell-based studies.

An essential next step is to explore PNA constructs targeting proven metabolic regulators such as PTP1B and PTEN. It will also be necessary to optimize tissue-directed cargo distribution via GLP-1R and GalNAc conjugation. Long-term safety data for PNA therapeutics must also be obtained, and scalable manufacturing methods for treating chronic diseases must be developed before clinical studies begin.

Addressing these challenges could enable a therapeutic strategy that integrates PNA-based gene expression control, CPP transport, and receptor-guided administration, thereby leading to the development of a new class of organ-directed therapies for T2D with fewer side effects. However, there is still a long way to go from preclinical concept to clinical translation.

No reviews have examined these three delivery platforms in parallel and in the context of metabolic disease. Identifying shared barriers to translation across these systems can support planning for future preclinical and early clinical investigations.

## Glossary

aCPPs: anionic cell-penetrating peptides
ADC: antibody-drug conjugates
ADCx: antibody–drug complexes
AP2: adaptor protein-2
ASGPR: asialoglycoprotein receptor
ASOs: antisense oligonucleotides
CpG: cytosine–phosphate–guanine
CPPs: cell-penetrating peptides
diLBA: dilactobionic acid
ENTPD3: ectonucleoside triphosphate diphosphohydrolase-3
Fab: fragment antigen-binding
FcRn: neonatal Fc receptor
FDA: Food and Drug Administration
GalNAc: N-acetylgalactosamine
GCGR: glucagon receptor
GET: glycosaminoglycan-binding enhanced transduction
GLP-1: glucagon-like peptide-1
GLP-1R: glucagon-like peptide-1 receptor
GLP-1RAs: glucagon-like peptide-1 receptor agonists
HAMA: human anti-mouse antibody
IgG: immunoglobulin G
IgM: immunoglobulin M
LNP: lipid nanoparticle
mAb: monoclonal antibody
mAbs: monoclonal antibodies
miRNAs: microRNAs
PEG: polyethylene glycol
PNAs: peptide nucleic acids
PPARγ: peroxisome proliferator-activated receptor gamma
PTEN: phosphatase and tensin homolog
PTP1B: protein tyrosine phosphatase 1B
RISC: RNA-induced silencing complex
RNase H: ribonuclease H
scFv: single-chain variable fragment
SGLT2: sodium–glucose cotransporter 2
siRNA: small interfering RNA
T2D: type 2 diabetes mellitus
TAT: trans-activator of transcription
tGalNAc: triantennary galactosamine
TLR9: Toll-like receptor 9
TMEM27: transmembrane protein 27
